# Tissue Microarray Analysis Reveals Heterogeneous Expression of Talin-1 and Lactate Dehydrogenase A in Non-small Cell Lung Cancer: Implications for Biomarker Reliability

**DOI:** 10.7759/cureus.105323

**Published:** 2026-03-16

**Authors:** Abduladim Hmmier, Paul Dowling

**Affiliations:** 1 Genetic Engineering, Libyan Biotechnology Research Center, Tripoli, LBY; 2 Clinical Proteomics Lab, Department of Biology, Faculty of Science and Engineering, Maynooth University, Maynooth, IRL

**Keywords:** biomarker utility, expression heterogeneity, immunohistochemistry (ihc), lactate dehydrogenase a (ldha), non-metastatic tumor, nsclc, talin-1, tmas, tumor stage and grade

## Abstract

Background

Tumour heterogeneity significantly impacts biomarker reliability in non-small cell lung cancer (NSCLC), such as lactate dehydrogenase A (LDHA) and Talin-1, thus complicating the validation of their diagnostic utility. This study investigated their expression heterogeneity in tissue microarrays (TMAs) from 40 non-metastatic NSCLC cases (24 squamous cell carcinomas, 16 adenocarcinomas) and 10 normal lung tissues, using standardised immunohistochemistry.

Methodology

Formalin-fixed, paraffin-embedded TMAs were stained with anti-LDHA and anti-Talin-1 antibodies. Cores were clustered based on their expression intensity, scored from 0 to 3 for intensity, and analysed against tumour grade/stage. A two-sided Pearson chi-square test was used to calculate the significance between normal and cancer tissue expression and to correlate the expression with the tumour grade/stage. Fisher’s exact test was also considered when nodule counts were less than five.

Results

LDHA expression was significantly higher in adenocarcinoma (χ² = 20.30, p < 0.001) and squamous cell carcinoma (χ² = 28.34, p < 0.001) compared with normal lung tissue, with a heterogeneous expression pattern, although no association was observed with tumour stage, grade, or lymph node status. These findings suggest that its expression heterogeneity may contribute to the inconsistency in its reported diagnostic utility across studies for lung cancer diagnosis, highlighting the need for multiplexed biomarker panels to overcome limitations driven by heterogeneity. In contrast, Talin-1 expression was reduced in malignancy and did not demonstrate statistical significance in adenocarcinoma (χ² = 2.11, p = 0.146), squamous cell carcinoma (χ² = 0.01, p = 0.912), or NSCLC overall (χ² = 1.12, p = 0.289). Talin-1 expression also had a heterogeneous pattern with no correlation with the tumour clinicopathological parameters.

Conclusions

Our findings revealed significant heterogeneity in LDHA and Talin-1 expression across NSCLC subtypes, independent of tumour grade and stage. This observed variability supports the need for using multiplexed panels and patient-based rather than single-marker approaches for reliable clinical applications.

## Introduction

Tumour heterogeneity and treatment outcomes

Tumour heterogeneity manifests at multiple levels, ranging from within individual tumours to between cancer subtypes and tissues of origin. This variability significantly influences immune-tumour interactions, underscoring the need to understand its role in shaping these critical biological relationships [1]. Highly heterogeneous tumours frequently demonstrate resistance to targeted therapies aimed at specific oncogenic drivers, thereby limiting their treatment efficacy. Conversely, molecularly targeted agents that induce partial tumour cell death may inadvertently increase heterogeneity and promote more aggressive phenotypes, further complicating therapeutic management by leading to the emergence of treatment-resistant cancer cells that can evade subsequent therapies [2]. Applied mathematical models suggest that optimised chemotherapy regimens, particularly those employing lower dose rates, may prove more effective against tumours containing resistant subpopulations [3]. The contribution of intra-tumour heterogeneity to drug resistance highlights the necessity of incorporating evolutionary principles into clinical trial design and therapeutic development [4].

Advances in tumour sampling and analysis

Obtaining representative tumour samples remains crucial for treatment planning. Current biopsy techniques, including core needle, fine-needle aspiration, laparoscopic/thoracoscopic, and surgical approaches (incision/excision), are selected based on the overall clinical context, tumour location, and patient status [5]. Image-guided biopsies, either guided by ultrasound, CT, MRI, or fusion imaging, have enhanced diagnostic accuracy through precise tumour targeting [6]. Recent innovations have now enabled concurrent metabolomic and histologic co-evaluations for a single biopsy specimen, maximising the information yield from limited tissue samples [7]. However, immunohistochemical (IHC) analysis continues to face challenges due to pre-analytical variability in sample handling and processing [8]. Despite being the gold standard for malignancy confirmation, IHC suffers from reproducibility issues stemming from inconsistent staining protocols and the lack of standardised fixation and antigen retrieval methods [9]. Variability in both pre-analytical procedures and post-analytical interpretation, particularly in threshold determination, contributes significantly to these inconsistencies [10]. Addressing these limitations through standardised protocols is essential for improving diagnostic accuracy [11].

Challenges in serological biomarker development

The development of non-invasive biomarkers for treatment monitoring and relapse detection faces substantial obstacles due to tumour heterogeneity [12]. Even histologically similar tumours from the same organ often exhibit divergent molecular profiles and therapeutic responses [13]. This genetic variability complicates the identification of biomarkers with excellent sensitivity and specificity for clinical use [14]. While current biomarkers may show limited overall sensitivity, certain markers could have utility in specific patient subsets, particularly those with distinct molecular profiles or specific tumour characteristics that influence treatment response [15]. The observed variability in biomarker performance across study populations primarily reflects inter-tumour heterogeneity [16], which can produce inconsistent expression patterns and weak biomarker outcome correlations [17]. Overcoming these challenges requires careful pilot studies to characterise heterogeneity patterns and strict biomarker reporting standards to ensure study transparency and reproducibility.

Aim of the study

This study aimed to characterise protein expression heterogeneity in tissue microarrays (TMAs) derived from non-metastatic non-small cell lung cancer (NSCLC) specimens with documented tumour grades and TNM stages. Expression patterns of lactate dehydrogenase A (LDHA) and Talin-1 were examined both within and between tumour nodules to assess variability and explore potential associations with tumour grade and stage. The objective was not to validate LDHA or Talin-1 as diagnostic or prognostic biomarkers but to illustrate how intra-tumour and inter-tumour heterogeneity may influence biomarker reliability and limit their clinical utility in NSCLC diagnosis.

This article was posted as a preprint on Research Square in 2026 under ISSN 2693-5015 (online) with the same title: Tissue Microarray Analysis Reveals Heterogeneous Expression of Talin-1 and Lactate Dehydrogenase A in Non-Small Cell Lung Cancer: Implications for Biomarker Reliability.

## Materials and methods

Experimental design

This study investigates the inconsistent expression patterns of Talin-1 and LDHA in non-metastatic NSCLC, focusing on both intra-tumour and inter-tumour heterogeneity. The purpose was to illustrate how these spatial variations impact the clinical utility of Talin-1 and LDHA as potential clinical biomarkers for NSCLC diagnosis and monitoring. Talin-1 and LDHA were chosen based on their well-documented involvement in tumour progression and expansion. Talin-1 plays crucial roles in cell adhesion and migration, while LDHA is a key enzyme in tumour metabolism through aerobic glycolysis. Both proteins have shown dysregulated expression in various cancers, including NSCLC. Our selection was further supported by preliminary findings from our bronchoalveolar lavage fluid analysis published earlier in 2017, which suggested their potential diagnostic value in NSCLC [18]. Utilising TMAs containing non-metastatic NSCLC samples, we conducted a systematic analysis of protein expression patterns across tumour nodules from different patients. This approach allowed us to assess the degree of heterogeneity in Talin-1 and LDHA expression and investigate their relationship with the clinical parameters, such as tumour grade and stage. The study design specifically addresses how tumour heterogeneity contributes to the observed inconsistency of these otherwise promising biomarkers in clinical applications.

TMA processing and IHC

The study utilised two commercially available NSCLC TMAs (LC10011b series from TissueArrays.com LLC) containing duplicate cores from 40 non-metastatic NSCLC cases (24 squamous cell carcinoma and 16 adenocarcinoma), along with 10 normal lung tissue controls. All IHC staining was performed at the National Institute for Cellular Biotechnology at Dublin City University (NICB-DCU) using a Dako automated stainer.

Primary antibodies against Talin-1 (C45F1 rabbit mAb #4021) and LDHA (C4B5 rabbit mAb #3582) were purchased from Cell Signalling Technology and used at manufacturer-recommended dilutions of 1:50 and 1:400, respectively. Tissue sections underwent antigen retrieval in citrate buffer at pH 6 for 20 minutes, followed by a 30-minute primary antibody incubation. Haematoxylin was used as a counterstain to visualise nuclei, with subsequent dehydration through an ethanol series and xylene before applying the coverslips.

Stained sections were evaluated under 20× magnification using a four-category scoring system: negative (0), weak (1), moderate (2), or strong (3). The scoring summary by tissue type and antibody is summarised in the Results section, and the full scoring results are endorsed in the Appendices, with representative examples shown in the Results section. Duplicate cores from each case were analysed independently to assess staining consistency across tissue samples.

Statistical analysis

Statistical analysis was performed to assess the association between IHC staining intensity and clinicopathological parameters. Staining intensity was scored on a four-point scale (0-3). Tumour stage was classified into early stage (stages I-II) and advanced stage (stage III) to ensure an adequate nodule count for significance analysis. Tumour grade was grouped into low grade (grades 1 and 2) and high grade (grades 2-3 and 3). TNM staging was utilised to compare the expression versus the localised and/or lymph node involvement. Associations between staining intensity and tumour stage or tumour grade were evaluated using the Pearson chi-square test. When expected cell counts were less than five, Fisher’s exact test was considered. All statistical tests were two-sided, and a p-value of less than 0.05 was considered statistically significant.

## Results

LDHA expression patterns across NSCLC subtypes

We examined LDHA expression patterns in relation to clinical staging across different NSCLC subtypes. In normal lung tissues (N), including two cases with pulmonary oedema (PO), all 20 cores demonstrated limited LDHA expression, with eight showing very weak staining (score 0) and 12 weak staining (score 1). No normal tissue exhibited moderate or strong staining (Table 1).

**Table 1:** Tissue microarray scoring. *: Staining failure in Talin-1 (three AD and one SqCC cores). Data were presented as the percentage of n/N, where n is the number of nodules that hit the specified score, and N is the total number of cores per NSCLC subtype. LT: normal lung tissue; AD: lung adenocarcinoma; SqCC: lung squamous cell carcinoma; LDHA: lactate dehydrogenase A; NSCLC: non-small cell lung cancer

Biomarker	Tissue type	Score			
		0	1	2	3
LDHA	Normal LT	8/20 (40%)	12/20 (60%)	0/20 (0%)	0/20 (0%)
	AD	3/32 (9.4%)	9/32 (28.1%)	16/32 (50%)	4/32 (12.5%)
	SqCC	4/48 (8.3%)	10/48 (20.8%)	20/48 (39.5%)	14/48 (31.25%)
Talin-1*	Normal LT	7/20 (35%)	9/20 (45%)	2/20 (10%)	2/20 (10%)
	AD	1/32 (3.1%)	15/32 (48.9%)	12/32 (37.5%)	1/32 (6.3%)
	SqCC	8/48 (16.7%)	27/48 (56.3%)	11/48 (23%)	1/48 (2%)

In adenocarcinoma and squamous cell carcinoma samples, LDHA expression varied across tumour stages (Table 2). Most adenocarcinoma cores showed moderate staining, with strong staining observed exclusively in stage IIIA tumours. squamous cell carcinoma samples demonstrated a broader distribution of staining intensities, ranging from negative to strong expression across stages IA-IIIB, with strong staining predominantly observed in stage IIIA tumours (Table 2). Two additional large cell lung cancer cores (stage IIA) showed weak staining, but they were not included in the final analysis.

**Table 2:** Correlation of LDHA and Talin-1 expression with tumour stage, grade, and lymph node involvement. Low intensity: score 0-1; high intensity: score 2-3. Low grade: grades 1 and 2. High grade: grades 2-3 and 3. No lymph involvement: TNM: T could be 1 to 3 nodules, N 0, M 0. Lymph nodes involved: T could be 1 to 3 nodules, N could be 1 to 3 nodules, and M 0. LDHA: lactate dehydrogenase A; AD: lung adenocarcinoma; SqCC: lung squamous cell carcinoma

Biomarker	Tumour type	Intensity	Tumour stage		Grade		TNM staging	
			Early (IA + IIA + IIB)	Advanced (IIIA + IIIB)	Low	High	No lymph node involved	Lymph nodes involved
LDHA	AD	Low	6	5	8	3	2	9
		High	11	11	16	7	2	18
	SqCC	Low	7	4	9	3	2	9
		High	15	18	21	13	2	32
Talin-1	AD	Low	10	6	11	5	3	13
		High	7	6	9	4	1	12
	SqCC	Low	15	15	26	8	2	27
		High	5	7	7	5	2	11

For statistical analysis, tumour stages were grouped into early (I-II) and advanced (III) categories (Table 2). Chi-square analysis demonstrated no statistically significant association between LDHA staining intensity and tumour stage in either adenocarcinoma (χ² = 6.13, p = 0.106) or squamous cell carcinoma (χ² = 1.34, p = 0.720). Fisher’s exact test similarly showed no significant relationship between LDHA expression and tumour grade in adenocarcinoma (p = 1.000) or squamous cell carcinoma (p = 0.31), nor with lymph node status in adenocarcinoma (p = 0.60) or squamous cell carcinoma (p = 0.52) (Table 2).

**Figure 1:**
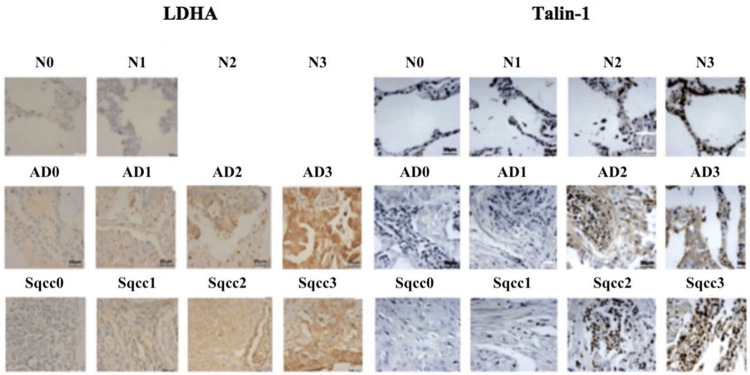
Immunohistochemistry analysis of LDHA and Talin-1 in NSCLC TMAs. TMAs, to detect the impact of tumour stage/grade on LDHA and Talin-1 level of expression and to know at what stage of non-metastatic cancer LDHA and Talin-1 are hugely produced. Further work was conducted on LDHA and Talin-1 in NSCLC TMAs for better consideration when evaluating their clinical utility.  Scoring: 0 (negative), 1 (weak), 2 (moderate), 3 (strong). The scoring system used is per tissue type (normal n = 20 cores from duplicates of 10 non-cancer individuals (two of which with pulmonary oedema); AD: n = 32 cores from duplicates of 16 AD patients (one of which with necrosis); Sqcc: n = 48 cores from duplicates of 24 patients with Sqcc (one of which with sparse). The scoring system was 0: no/very weak staining, 1: weakly stained, 2: moderate staining, 3: strong staining. LDHA: lactate dehydrogenase A; N: normal lung tissue; AD: adenocarcinoma; Sqcc: squamous cell carcinoma; TMA: tissue microarray; NSCLC: non-small cell lung cancer

Overall, these findings indicate no clear correlation between LDHA expression intensity and tumour stage, grade, or nodal involvement, with substantial variability observed within each stage category. Complete scoring details are provided in the Appendices, with representative cores shown in Figure 1 (left panel).

However, when evaluated as a diagnostic marker, LDHA expression differed significantly between normal lung tissue and tumour samples. Pearson’s chi-square analysis demonstrated significantly higher LDHA staining in adenocarcinoma compared with normal tissue (χ² = 20.30, p < 0.001), and a similar association was observed for squamous cell carcinoma (χ² = 28.34, p < 0.001). When all NSCLC samples were analysed collectively, LDHA expression remained significantly elevated compared with normal tissue (χ² = 29.40, p < 0.001) (Table 3). These findings suggest that LDHA overexpression is strongly associated with malignant lung tissue and may serve as a potential diagnostic biomarker distinguishing non-metastatic NSCLC from normal lung tissue.

**Table 3:** Comparison of LDHA and Talin-1 expression in normal and NSCLC. Intensity: expression of the tested biomarkers. Low intensity: score 0-1. High intensity: score 2-3. LDHA: lactate dehydrogenase A; Normal: normal lung tissue; AD: lung adenocarcinoma; SqCC: lung squamous cell carcinoma; NSCLC: non-small cell lung cancer

Biomarker	Intensity	Normal	AD	SqCC	NSCLC
LDHA	Low	20	11	14	25
	High	0	20	34	54
Talin-1	Low	16	16	35	51
	High	4	13	12	25

Talin-1 expression patterns in NSCLC subtypes

Figure 1 (right panel) illustrates Talin-1 immunohistochemical reactivity across TMA cores representing normal lung tissue (N), lung adenocarcinoma, and squamous cell carcinoma. In normal lung tissues (n = 20), including two cases with pulmonary oedema, Talin-1 expression was generally low, with most cores demonstrating negative or weak staining and only occasional moderate or strong reactivity (Table 1).

Adenocarcinoma samples (n = 32) displayed variable Talin-1 expression across tumour stages (Tables 1, 2). Most cores demonstrated weak to moderate staining, while strong reactivity was rare and observed in only a single stage IIIA tumour. Three cores could not be evaluated due to technical difficulties.

Squamous cell carcinoma samples (n = 48) showed more heterogeneous staining patterns, ranging from negative to moderate expression across stages IA-IIIB, with strong staining observed in only one stage IIB tumour. One core failed the technical evaluation (Tables 1, 2).

Statistical analysis demonstrated no significant association between Talin-1 staining intensity and tumour stage in either adenocarcinoma (χ² = 3.23, p = 0.357) or squamous cell carcinoma (χ² = 2.41, p = 0.492). Fisher’s exact test also revealed no significant relationship between Talin-1 expression and tumour grade in adenocarcinoma (p = 1.000) or squamous cell carcinoma (p = 0.47), nor with lymph node status in adenocarcinoma (p = 0.62) or squamous cell carcinoma (p = 0.24). Overall, Talin-1 expression showed no consistent correlation with tumour stage, grade, or nodal involvement (Table 2). Complete scoring details are provided in the Appendices.

When Talin-1 expression was compared between normal lung tissue and NSCLC subtypes, no statistically significant differences were observed after grouping staining intensity into low (0-1) and high (2-3) categories (Table 3). There was no significant association between normal tissue and adenocarcinoma (χ² = 2.11, p = 0.146) or squamous cell carcinoma (χ² = 0.01, p = 0.912). Similarly, when all tumour cases were analysed collectively, the difference remained non-significant (χ² = 1.12, p = 0.289), indicating that Talin-1 expression does not markedly differ between malignant and normal lung tissues.

## Discussion

Clinical utility of LDHA expression in NSCLC

In this study, LDHA expression was highly abundant in malignant lung tissues in both adenocarcinoma and squamous cell carcinoma compared with normal lung samples (p < 0.001). This diagnostic association aligns with the previous research demonstrating metabolic reprogramming as a hallmark of cancer, in which LDHA facilitates aerobic glycolysis and supports tumour cell proliferation, survival, and adaptation to hypoxia [19,20]. Increased LDHA expression has been reported in multiple malignancies, including NSCLC, and is frequently linked to poor clinical outcomes and enhanced tumour aggressiveness [21,22].

Despite its diagnostic potential, LDHA did not show a significant correlation with tumour stage, grade, or nodal involvement in our cohort. This suggests that LDHA overexpression may represent an early event in malignant transformation rather than a marker of progression or aggressiveness [20]. Previous investigations have similarly reported that glycolytic enzyme upregulation can occur across tumour stages without a stage-dependent pattern, reflecting metabolic plasticity rather than anatomical progression [19]. In NSCLC, dynamic metabolic shifts appear to precede morphological advancement, supporting the concept that metabolic markers may be more sensitive for early detection than stage stratification [20,22].

The observed intra-stage heterogeneity in LDHA expression underscores the influence of tumour microenvironmental factors, such as hypoxia and regional nutrient gradients, on metabolic programming [23,24]. As a result, LDHA may have greater utility as a diagnostic biomarker distinguishing neoplastic from non-neoplastic tissue rather than as a prognostic indicator of tumour progression [19,21].

Talin-1 expression and the complexity of cytoskeletal biomarkers

Talin-1 is a focal adhesion protein that connects integrins to the actin cytoskeleton and modulates cell adhesion, migration, and mechano-transduction [25,26]. Unlike LDHA, Talin-1 expression was similar between normal lung tissue and NSCLC subtypes and, like LDHA, did not correlate with tumour stage, grade, or lymph node status. Dysregulated adhesion signalling has been implicated in cancer cell invasion and metastasis; however, static IHC expression levels may not capture the functional dynamics associated with tumour progression [25,26]. Several studies have shown that the prognostic value of cytoskeletal and adhesion proteins such as Talin-1 may lie in post-translational modifications, subcellular distribution, or interactions with integrin complexes rather than absolute expression levels [26,27].

The lack of an obvious pattern in Talin-1 expression may also reflect tumour heterogeneity and the influence of stromal elements, extracellular matrix composition, or differential integrin repertoires among tumour cells [27]. Such complexity makes interpretation challenging when using single-marker IHC quantification, in line with observations from other studies evaluating adhesion-related proteins in solid tumours [26-28].

Biomarker reliability and tumour heterogeneity

The heterogeneous profiles of LDHA and Talin-1 expression patterns illustrate fundamental challenges in biomarker research. Heterogeneity in tumour biology manifests at multiple levels: genetic, epigenetic, metabolic, and microenvironmental, leading to variability in IHC staining patterns even within the same histological stage [29,30]. This complexity reduces the sensitivity and specificity of single biomarkers when used as stand-alone indicators for prognostic stratification or staging correlation [29]. Prior research in NSCLC has highlighted the limitations of relying solely on tissue biomarkers for clinical decision-making due to spatial, temporal, and cellular heterogeneity [30]. These studies emphasise the need for integrative approaches that combine histopathology with other molecular dimensions, such as gene expression signatures, circulating biomarkers, and advanced imaging modalities [30].

The value of formalin-fixed, paraffin-embedded tissues and TMA platforms

TMAs constructed from formalin-fixed, paraffin-embedded (FFPE) specimens remain invaluable for high-throughput biomarker validation [29,30]. Advances in antigen retrieval, multiplexed IHC, and proteomic extraction have resolved many historical challenges associated with FFPE analysis, enabling reliable quantification of protein expression from archived materials [29,30]. The extensive availability of FFPE lung cancer specimens with well-annotated clinical data offers opportunities for large-scale retrospective validation and cross-cohort comparisons as essential steps towards clinical translation of candidate biomarkers [29,30].

Summary

The reliability of lung cancer biomarkers continues to face significant challenges, primarily due to three key factors: variability in study methodologies, heterogeneity in patient populations, and inconsistencies in results interpretation. This problem proves particularly complex in adenocarcinomas, where many diagnostic biomarkers simultaneously serve prognostic functions, creating potential conflicting effects in clinical interpretation. The dual diagnostic-prognostic nature of these markers necessitates careful validation across diverse clinical settings to establish their true utility. Emerging approaches in biomarker discovery, including metabolomic profiling, liquid biopsy technologies, and advanced proteomic analysis of FFPE tissues, show promise in overcoming these limitations. The persistent heterogeneity observed in markers such as LDHA and Talin-1 underscores the need for standardised, multiplexed detection strategies that account for spatial and temporal variations in tumour biology. However, similar findings have reported such heterogeneity in various metabolomic and cytoskeletal-based biomarkers in NSCLC, where both intra- and inter-tumour expression variance limit their diagnostic sensitivity and prognostic utility when considered for single time point diagnosis. Studies evaluating the clinical utility of protein biomarkers for early detection, patient stratification, and treatment, multiple reaction monitoring (MRM) consistently have shown that spatial heterogeneity, microenvironmental influence, and dynamic metabolic reprogramming can prevent correlating them with tumour stage, grade, and/or clinical outcome, reducing their reliability as stand-alone diagnostic or surveillance markers. Consequently, both LDHA and Talin-1 explained the challenges encountered in translating tissue-based biomarkers into clinically actionable markers for cancer diagnosis and MRM. Moving forward, integrating multi-omics data with clinically annotated FFPE repositories will be critical for developing robust, clinically actionable biomarkers that improve NSCLC diagnosis, prognosis, and personalised therapeutic strategies.

Future directions

Even though LDHA shows promise as a marker for distinguishing cancerous from normal lung tissue, its ability to predict outcomes is limited because tumours can differ greatly from one to another. Future investigations should integrate LDHA expression with comprehensive metabolic profiling, hypoxia markers, and genomic signatures to better characterise its clinical significance. Multi-omics approaches that bridge proteomics with transcriptomics and metabolomics may yield more robust and clinically actionable biomarkers, particularly when combined with LDHA expression data and other relevant clinical parameters.

For Talin-1 and other adhesion-related proteins, functional studies exploring subcellular localisation, phosphorylation states, and interactive network dynamics may offer deeper insights than static IHC expression alone. Additionally, incorporating machine learning models that combine multiple markers with clinical and imaging data could improve diagnostic and prognostic accuracy in NSCLC.

However, there is a need to implement standardised protocols in biomarker discovery and to initiate a central tele-biomarker discovery platform, which would allow all biomarker discovery research to be simultaneously uploaded to a worldwide platform, enabling experts to share their final judgement on biomarker validation and offering a more approachable and non-invasive strategy to make biomarker discovery in cancer more realistic.

## Conclusions

Our findings reveal significant heterogeneity in LDHA and Talin-1 expression across NSCLC subtypes, independent of tumour grade/stage. This underscores the need for standardised IHC protocols and spatial profiling in biomarker development. The variability observed supports the need for using multiplexed panels rather than single-marker approaches for reliable clinical applications.

## Appendices

**Table 4:** TMA: relevant clinical data of core tissue samples including stage and grade and their scores for LDHA and Talin-1 expression. TMA: tissue microarray; LDHA: lactate dehydrogenase A

Position	Number	Age	Sex	Organ/Anatomic site	Pathology diagnosis	TNM	Grade	Stage	Type	Tissue ID	LDH score	Talin 1 score
G2	62	56	M	Lung	Adenocarcinoma	T2N1M0	2	IIB	Malignant	Rln060557	0	2
F3	53	51	M	Lung	Adenocarcinoma	T3N1M0	2--3	IIIA	Malignant	Rln060214	0	x
J2	92	47	M	Lung	Lung tissue	-	-	-	Normal	Rln06N004	0	0
J3	93	49	M	Lung	Lung tissue	-	-	-	Normal	Rln04N006	0	0
J4	94	24	M	Lung	Lung tissue	-	-	-	Normal	Rln03N010	0	0
I5	85	21	F	Lung	Lung tissue	-	-	-	Normal	Rln06N024	0	1
J8	98	49	M	Lung	Lung tissue	-	-	-	Normal	Rln04N006	0	1
I3	83	48	M	Lung	Lung tissue	-	-	-	Normal	Rln05N023	0	2
I8	88	48	M	Lung	Lung tissue	-	-	-	Normal	Rln05N023	0	2
I10	90	21	F	Lung	Lung tissue	-	-	-	Normal	Rln06N024	0	3
C7	27	50	M	Lung	Squamous cell carcinoma	T2N2M0	2	IIIA	Malignant	Rln100083	0	1
D1	31	50	M	Lung	Squamous cell carcinoma	T1N0M0	2	IA	Malignant	189 Rln A1	0	1
C2	22	50	M	Lung	Squamous cell carcinoma (sparse)	T2N2M0	*	IIIA	Malignant	Rln100083	0	0
C8	28	52	M	Lung	Squamous cell carcinoma (sparse)	T2N2M0	2	IIIA	Malignant	Rln060265	0	2
F4	54	54	M	Lung	Adenocarcinoma	T3N1M0	2	IIIA	Malignant	Rln060359	1	1
G5	65	68	M	Lung	Adenocarcinoma	T2N1M0	2--3	IIB	Malignant	Rln060217	1	1
H1	71	64	M	Lung	Adenocarcinoma	T2N2M0	2	IIIA	Malignant	Rln130059	1	1
F8	58	51	M	Lung	Adenocarcinoma	T3N1M0	2	IIIA	Malignant	Rln060214	1	2
G7	67	56	M	Lung	Adenocarcinoma	T2N1M0	2	IIB	Malignant	Rln060557	1	2
H6	76	64	M	Lung	Adenocarcinoma	T2N2M0	2	IIIA	Malignant	Rln130059	1	2
F1	51	67	F	Lung	Adenocarcinoma	T2N1M0	2--3	IIB	Malignant	Rln100022	1	x
H5	75	54	M	Lung	Adenocarcinoma with necrosis	T2N0M0	3	IIA	Malignant	Rln050361	1	1
H10	80	54	M	Lung	Adenocarcinoma with necrosis	T2N0M0	3	IIA	Malignant	Rln050361	1	2
I4	84	19	M	Lung	Lung tissue	-	-	-	Normal	Rln06N027	1	0
J5	95	35	M	Lung	Lung tissue	-	-	-	Normal	Rln06N013	1	0
I1	81	2	F	Lung	Lung tissue	-	-	-	Normal	Rln08N035	1	1
I2	82	33	M	Lung	Lung tissue	-	-	-	Normal	Rln07N026	1	1
I6	86	2	F	Lung	Lung tissue	-	-	-	Normal	Rln08N035	1	1
I7	87	33	M	Lung	Lung tissue	-	-	-	Normal	Rln07N026	1	1
J7	97	47	M	Lung	Lung tissue	-	-	-	Normal	Rln06N004	1	1
J9	99	24	M	Lung	Lung tissue	-	-	-	Normal	Rln03N010	1	1
J10	100	35	M	Lung	Lung tissue	-	-	-	Normal	Rln06N013	1	1
I9	89	19	M	Lung	Lung tissue	-	-	-	Normal	Rln06N027	1	3
J1	91	18	F	Lung	Lung tissue (pulmonary oedema)	-	-	-	Normal	Rln06N010	1	0
J6	96	18	F	Lung	Lung tissue (pulmonary oedema)	-	-	-	Normal	Rln06N010	1	0
B3	13	56	M	Lung	Squamous cell carcinoma	T2N3M0	2	IIIB	Malignant	Rln100093	1	0
B8	18	56	M	Lung	Squamous cell carcinoma	T2N3M0	2	IIIB	Malignant	Rln100093	1	0
D6	36	50	M	Lung	Squamous cell carcinoma	T1N0M0	*	IA	Malignant	189 Rln A1	1	0
D7	37	61	M	Lung	Squamous cell carcinoma	T2N1M0	2	IIB	Malignant	182800B15	1	0
A3	3	60	M	Lung	Squamous cell carcinoma	T2N1M0	2	IIB	Malignant	1144C2	1	1
B1	11	58	M	Lung	Squamous cell carcinoma	T2N1M0	*	IIB	Malignant	Rln060220	1	1
B6	16	58	M	Lung	Squamous cell carcinoma	T2N1M0	2	IIB	Malignant	Rln060220	1	1
B7	17	60	M	Lung	Squamous cell carcinoma	T2N3M0	2	IIIB	Malignant	Rln100089	1	1
D3	33	61	M	Lung	Squamous cell carcinoma	-	3	-	Malignant	1972C1	1	1
E1	41	36	F	Lung	Squamous cell carcinoma	T2N1M0	2	IIB	Malignant	Rln060595	1	1
E5	45	69	M	Lung	Adenocarcinoma	T2N2M0	2	IIIA	Malignant	Rln100012	2	0
F5	55	63	F	Lung	Adenocarcinoma	T2N2M0	2	IIIA	Malignant	Rln060503	2	1
F6	56	67	F	Lung	Adenocarcinoma	T2N1M0	*	IIB	Malignant	Rln100022	2	1
G1	61	59	M	Lung	Adenocarcinoma	T2N1M0	2--3	IIB	Malignant	Rln060372	2	1
G4	64	66	F	Lung	Adenocarcinoma	T2N1M0	3	IIB	Malignant	Rln110015	2	1
G6	66	59	M	Lung	Adenocarcinoma	T2N1M0	2	IIB	Malignant	Rln060372	2	1
G8	68	67	M	Lung	Adenocarcinoma	T2N1M0	*	IIB	Malignant	Rln100030	2	1
H2	72	68	M	Lung	Adenocarcinoma	T3N0M0	2	IIB	Malignant	Rln030512	2	1
H4	74	71	M	Lung	Adenocarcinoma	T2N1M0	3	IIB	Malignant	182645B15	2	1
H7	77	68	M	Lung	Adenocarcinoma	T3N0M0	2	IIB	Malignant	Rln030512	2	1
E10	50	69	M	Lung	Adenocarcinoma	T2N2M0	2	IIIA	Malignant	Rln100012	2	2
F9	59	54	M	Lung	Adenocarcinoma	T3N1M0	2	IIIA	Malignant	Rln060359	2	2
F10	60	63	F	Lung	Adenocarcinoma	T2N2M0	2	IIIA	Malignant	Rln060503	2	2
G3	63	67	M	Lung	Adenocarcinoma	T2N1M0	*	IIB	Malignant	Rln100030	2	2
G9	69	66	F	Lung	Adenocarcinoma	T2N1M0	3	IIB	Malignant	Rln110015	2	2
H9	79	71	M	Lung	Adenocarcinoma	T2N1M0	3	IIB	Malignant	182645B15	2	2
B2	12	60	M	Lung	Squamous cell carcinoma	T2N3M0	1	IIIB	Malignant	Rln100089	2	0
D2	32	61	M	Lung	Squamous cell carcinoma	T2N1M0	2	IIB	Malignant	182800B15	2	0
D5	35	66	M	Lung	Squamous cell carcinoma	T3N1M0	3	IIIA	Malignant	Rln050466	2	0
A1	1	69	M	Lung	Squamous cell carcinoma	T2N1M0	2	IIB	Malignant	Rln070008	2	1
A2	2	44	M	Lung	Squamous cell carcinoma	T2N2M0	2	IIIA	Malignant	Rln100101	2	1
A4	4	37	M	Lung	Squamous cell carcinoma	T2N1M0	2	IIB	Malignant	66237C1	2	1
A6	6	69	M	Lung	Squamous cell carcinoma	T2N1M0	2	IIB	Malignant	Rln070008	2	1
A7	7	44	M	Lung	Squamous cell carcinoma	T2N2M0	2	IIIA	Malignant	Rln100101	2	1
A8	8	60	M	Lung	Squamous cell carcinoma	T2N1M0	2	IIB	Malignant	1144C2	2	1
A9	9	37	M	Lung	Squamous cell carcinoma	T2N1M0	*	IIB	Malignant	66237C1	2	1
A10	10	64	M	Lung	Squamous cell carcinoma	T2N1M0	2	IIB	Malignant	Rln110014	2	1
B4	14	71	M	Lung	Squamous cell carcinoma	T2N2M0	2	IIIA	Malignant	Rln050374	2	1
C1	21	69	M	Lung	Squamous cell carcinoma	T2N2M0	2	IIIA	Malignant	Rln050456	2	1
C5	25	63	M	Lung	Squamous cell carcinoma	T3N1M0	2--3	IIIA	Malignant	Rln050439	2	1
D8	38	61	M	Lung	Squamous cell carcinoma	-	3	-	Malignant	1972C1	2	1
A5	5	64	M	Lung	Squamous cell carcinoma	T2N1M0	2	IIB	Malignant	Rln110014	2	2
B9	19	71	M	Lung	Squamous cell carcinoma	T2N2M0	2	IIIA	Malignant	Rln050374	2	2
C10	30	63	M	Lung	Squamous cell carcinoma	T3N1M0	2--3	IIIA	Malignant	Rln050439	2	2
E6	46	36	F	Lung	Squamous cell carcinoma	T2N1M0	3	IIB	Malignant	Rln060595	2	2
D10	40	66	M	Lung	Squamous cell carcinoma	T3N1M0	3	IIIA	Malignant	Rln050466	2	?
H3	73	61	F	Lung	Adenocarcinoma	T3N1M0	2	IIIA	Malignant	Rln130002	3	1
H8	78	61	F	Lung	Adenocarcinoma	T3N1M0	2	IIIA	Malignant	Rln130002	3	1
F7	57	52	M	Lung	Adenocarcinoma	T2N2M0	1	IIIA	Malignant	Rln110046	3	3
F2	52	52	M	Lung	Adenocarcinoma	T2N2M0	2	IIIA	Malignant	Rln110046	3	x
B5	15	52	M	Lung	Squamous cell carcinoma	T2N2M0	2	IIIA	Malignant	Rln060199	3	1
C6	26	69	M	Lung	Squamous cell carcinoma	T2N2M0	2	IIIA	Malignant	Rln050456	3	1
D4	34	41	M	Lung	Squamous cell carcinoma	T2N2M0	3	IIIA	Malignant	Rln050382	3	1
D9	39	41	M	Lung	Squamous cell carcinoma	T2N2M0	*	IIIA	Malignant	Rln050382	3	1
E3	43	37	M	Lung	Squamous cell carcinoma	T2N1M0	3	IIB	Malignant	66237C5	3	1
E4	44	58	M	Lung	Squamous cell carcinoma	T2N1M0	3	IIB	Malignant	Rln060354	3	1
E8	48	37	M	Lung	Squamous cell carcinoma	T2N1M0	3	IIB	Malignant	66237C5	3	1
B10	20	52	M	Lung	Squamous cell carcinoma	T2N2M0	2	IIIA	Malignant	Rln060199	3	2
C3	23	52	M	Lung	Squamous cell carcinoma	T2N2M0	2	IIIA	Malignant	Rln060265	3	2
C9	29	65	M	Lung	Squamous cell carcinoma	T3N0M0	2	IIB	Malignant	Rln050414	3	2
E2	42	58	M	Lung	Squamous cell carcinoma	T2N2M0	3	IIIA	Malignant	Rln100086	3	2
E7	47	58	M	Lung	Squamous cell carcinoma	T2N2M0	3	IIIA	Malignant	Rln100086	3	2
E9	49	58	M	Lung	Squamous cell carcinoma	T2N1M0	3	IIB	Malignant	Rln060354	3	2
C4	24	65	M	Lung	Squamous cell carcinoma	T3N0M0	*	IIB	Malignant	Rln050414	3	3
G10	70	68	M	Lung	Adenocarcinoma	T2N1M0	3	IIB	Malignant	Rln060217	?	2

## References

[1] Knoche SM, Larson AC, Sliker BH, Poelaert BJ, Solheim JC (2021). The role of tumor heterogeneity in immune-tumor interactions. Cancer Metastasis Rev.

[2] Ramón Y Cajal S, Sesé M, Capdevila C (2020). Clinical implications of intratumor heterogeneity: challenges and opportunities. J Mol Med (Berl).

[3] Kozłowska E, Suwiński R, Giglok M, Świerniak A, Kimmel M (2020). Mathematical model predicts response to chemotherapy in advanced non-resectable non-small cell lung cancer patients treated with platinum-based doublet. PLoS Comput Biol.

[4] Venkatesan S, Swanton C (2016). Tumor evolutionary principles: how intratumor heterogeneity influences cancer treatment and outcome. Am Soc Clin Oncol Educ Book.

[5] You C, Li X, Du Y, Peng L, Wang H, Zhang X, Wang A (2022). The microultrasound-guided prostate biopsy in detection of prostate cancer: a systematic review and meta-analysis. J Endourol.

[6] O'Shea A, Tam AL, Kilcoyne A, Flaherty KT, Lee SI (2021). Image-guided biopsy in the age of personalised medicine: strategies for success and safety. Clin Radiol.

[7] Capalbo E, Peli M, Lovisatti M, Cosentino M, Mariani P, Berti E, Cariati M (2014). Trans-thoracic biopsy of lung lesions: FNAB or CNB? Our experience and review of the literature. Radiol Med.

[8] Marhana IA, Widianiti K, Kusumastuti EH (2022). Conformity of fine needle aspiration biopsy (FNAB) and core needle biopsy (CNB) in peripheral lung tumor patients: a cross-sectional study. Ann Med Surg (Lond).

[9] Deng H (2024). Utility of immunohistochemistry in the diagnosis of pleuropulmonary and mediastinal cancers: a review and update. Arch Pathol Lab Med.

[10] Zhu CQ, Tsao MS (2014). Prognostic markers in lung cancer: is it ready for prime time?. Transl Lung Cancer Res.

[11] Miller RT (2019). Avoiding pitfalls in diagnostic immunohistochemistry-important technical aspects that every pathologist should know. Semin Diagn Pathol.

[12] Leong TY, Cooper K, Leong AS (2010). Immunohistology--past, present, and future. Adv Anat Pathol.

[13] Inamura K (2018). Update on immunohistochemistry for the diagnosis of lung cancer. Cancers (Basel).

[14] Lee CY, Kennedy BC, Richoz N (2023). Time-, tissue- and treatment-associated heterogeneity in tumour-residing migratory DCs. bioRxiv.

[15] Hoang CD (2017). Protein-based prognostic biomarkers in lung cancer: promise or pitfall?. J Thorac Cardiovasc Surg.

[16] Cusnir M, Cavalcante L (2012). Inter-tumor heterogeneity. Hum Vaccin Immunother.

[17] Passaro A, Al Bakir M, Hamilton EG (2024). Cancer biomarkers: emerging trends and clinical implications for personalized treatment. Cell.

[18] Hmmier A, O'Brien ME, Lynch V, Clynes M, Morgan R, Dowling P (2017). Proteomic analysis of bronchoalveolar lavage fluid (BALF) from lung cancer patients using label-free mass spectrometry. BBA Clin.

[19] Tufail M, Jiang CH, Li N (2024). Altered metabolism in cancer: insights into energy pathways and therapeutic targets. Mol Cancer.

[20] Wang S, Lv J, Lv J (2022). Prognostic value of lactate dehydrogenase in non-small cell lung cancer patients with brain metastases: a retrospective cohort study. J Thorac Dis.

[21] de la Cruz-López KG, Castro-Muñoz LJ, Reyes-Hernández DO, García-Carrancá A, Manzo-Merino J (2019). Lactate in the regulation of tumor microenvironment and therapeutic approaches. Front Oncol.

[22] Feng Y, Xiong Y, Qiao T, Li X, Jia L, Han Y (2018). Lactate dehydrogenase A: a key player in carcinogenesis and potential target in cancer therapy. Cancer Med.

[23] Mishra D, Banerjee D (2019). Lactate dehydrogenases as metabolic links between tumor and stroma in the tumor microenvironment. Cancers (Basel).

[24] Huang B, Shen W, Jia Y (2025). LDHAα, a lactate dehydrogenase A isoform, promotes glycolysis and tumor progression. FEBS J.

[25] Lu F, Zhu L, Bromberger T (2022). Mechanism of integrin activation by talin and its cooperation with kindlin. Nat Commun.

[26] Baster Z, Russell L, Rajfur Z (2025). A review of Talin- and Integrin-dependent molecular mechanisms in cancer invasion and metastasis. Int J Mol Sci.

[27] Zhao Y, Lykov N, Tzeng C (2022). Talin‑1 interaction network in cellular mechanotransduction (Review). Int J Mol Med.

[28] Sakamoto S, McCann RO, Dhir R, Kyprianou N (2010). Talin1 promotes tumor invasion and metastasis via focal adhesion signaling and anoikis resistance. Cancer Res.

[29] Parra-Medina R, Castañeda-González JP, Chaves-Cabezas V, Alzate JP, Chaves JJ (2024). Diagnostic performance of immunohistochemistry markers for malignant pleural mesothelioma diagnosis and subtypes. A systematic review and meta-analysis. Pathol Res Pract.

[30] Marusyk A, Almendro V, Polyak K (2012). Intra-tumour heterogeneity: a looking glass for cancer?. Nat Rev Cancer.

